# Investigating the role of clinical exposure on motivational self-regulation skills in medical students based on cognitive apprenticeship model

**DOI:** 10.1186/s12909-024-05253-0

**Published:** 2024-03-08

**Authors:** Mahla Salajegheh, Azadeh Rooholamini, Ali Norouzi

**Affiliations:** 1 Department of Medical Education, Medical Education Development Center, Kerman University of Medical Sciences, Kerman, Iran; 2 Education Development Center, School of Medicine, Zanjan University of Medical Sciences, Zanjan, Iran

**Keywords:** Meta motivation, Medical education, Clinical exposure, Cognitive apprenticeship model, Motivational self-regulation

## Abstract

**Background:**

The importance of motivation regulation in medical students is highly significant due to their unique educational circumstances, such as clinical exposure. However, the role of clinical exposure in learning motivational self-regulation skills in students has not been explored thus far. This current study aims to investigate the role of clinical exposure on motivational self-regulation skills in medical students based on cognitive apprenticeship model.

**Method:**

This study was descriptive-analytical research conducted in 2022 on medical students. Data collection involved two questionnaires including Meta motivational Strategies in Medical Students Questionnaire and Maastricht Clinical Teaching Questionnaire. The research comprised two stages including measuring motivational self-regulation strategies in students before entering the clinical exposure phase, and simultaneous measurement of clinical exposure based on the student’s viewpoint and their motivational self-regulation strategies at the end of the first term of clinical exposure.

**Results:**

The results revealed a significant relationship between six dimensions of the cognitive apprenticeship model, including modeling, coaching, scaffolding, reflection, exploration, and learning environment, with motivational self-regulation strategies. However, there was no significant relationship between the articulation dimension of the cognitive apprenticeship model and motivational self-regulation strategies.

**Conclusion:**

Clinical exposure indirectly enhances students’ metacognitive skills. Observing the behavior of clinical faculty in the clinical exposure setting leads to the improvement of motivational self-regulation strategies in medical students.

## Introduction

Successful students employ specific strategies to enhance their motivation [1] and consequently, improve their performance [2]. Regulating motivation involves students’ efforts to change or control their motivation to accomplish a task, which can be tiring or challenging [3]. Motivational regulation has positive effects on students’ effort, perseverance, and future performance [4].

In medical students, the regulation of motivation holds greater importance due to their unique educational circumstances, such as clinical education. Clinical education means training that includes patient experiences. This process may be performed in health care facilities, outpatient clinics, emergency centers, hospitals, or private offices, and under the supervision of a teaching staff [5]. Clinical education plays a crucial role in bridging the gap between theoretical knowledge and practical application, preparing students for the realities of clinical practice [6]. In a professional curriculum that prepares students for patient care, most of the learning happens during clinical practice experiences [7]. In many medical education systems, clinical education usually begins in the third or fourth year of medical school and continues through clerkships and internships. Considering the role of clinical education, the first exposure of medical students to the real clinical environment has more importance [8].

Based on Systems thinking theory, motivational self-regulation is a complex and multifaceted process which having multiple dimensions [9]. Systems thinking theory emphasizes looking at systems as interconnected and interdependent wholes, where various factors influence each other. So, when applied to the concept of motivational self-regulation, it implies that there are numerous interconnected factors and processes involved in regulating one’s motivation [10].

Therefore, the motivational self-regulation process is influenced by various factors in the clinical learning environment, where multiple role models are present [11], including biological, developmental, sociocultural, and individual differences, all of which impact medical students’ efforts in motivational regulation [12].

However, researchers in the field of motivational self-regulation often underestimate the importance of sociocultural factors in motivational self-regulation of students [13]. Socio-cultural factors, through various means such as modeling, scaffolding, exploration, and sociocultural processes, influence motivational regulation [13]. As a central principle in social-cognitive theories of learning and behavior, these factors are the most prominent and significant aspects in enhancing motivational self-regulation in medical students [14].

Given the clinical learning environment provides a diverse array of potential sociocultural factors for motivational regulation in medical students, the presence of a competent clinical faculty member plays a crucial role in successful clinical education [15]. Among the foundational theories guiding clinical faculty members in being role models, there is the cognitive apprenticeship model which consists of sociocultural factors. This model is rooted in situated learning theory, which views knowledge as dynamically constructed in social contexts and learning as a socially situated activity deeply intertwined with interaction in a structured environment [16]. It encompasses six dimensions modeling, coaching, scaffolding, articulation, reflection, and exploration [17]. Nevertheless, there is a lack of studies on the impact of clinical faculty, on students’ awareness and skills necessary for self-regulating their motivation [18].

Based on the aforementioned points, the present study focuses on two research questions:

1. What is the role of clinical exposure in the acquisition of motivational self-regulation strategies in medical students based on the cognitive apprenticeship model?
2. To what extent can instructional strategies in the cognitive apprenticeship model impact the motivational self-regulation strategies of medical students?

## Methods

### Design and setting

This study was descriptive-analytical research conducted in 2022 at Kerman University of Medical Sciences (KMU). At KMU, the first entrance of medical students in clinical education is Clinical Clerkship after the Basic Medical Sciences and Physiopathology stages. Those students who enter Clinical Clerkship, for 23 consecutive months, pass all hospital wards. In Clinical Clerkship, observing the approach to disorders and patient management is emphasized. A clinical daily activity plan for medical students in Clinical Clerkship at KMU includes patients’ visit updates and reviews, morning reports, clinical rounds, hospital training conferences, and ambulatory training.

### Participants and sampling

The population in this study consisted of medical students in the Clinical Clerkship phase at KMU. A total of 80 students were selected through a census sampling method.

### Tools/Instruments

To collect data on motivational self-regulation, we utilized the Meta motivational Strategies in Medical Students Questionnaire (MSMQ), which was designed and validated by Noroozi et al. (2021) [19]. The questionnaire comprised 28 items categorized into 7 domains. All items were assessed on a 5-point Likert scale ranging from “never” to “always”. The content validity of the questionnaire was confirmed with a content validity index of 0.79. The reliability of the questionnaire was confirmed with a Cronbach’s alpha coefficient of 0.89 and an intra-class correlation coefficient of 0.87.

The regulation of the value domain explored the students’ perspectives on considering the high value of medical education and its relevance to their future careers, as well as allocating more time to important and valuable subjects to control their academic motivation. The domain of environmental structuring focuses on creating a conducive and focused environment for studying. The domain of regulation of relatedness emphasized the importance of establishing connections with key individuals in the field of medical education, as it can enhance students’ motivation. The domain of promotional situational awareness examined the students’ opinions on obtaining information about their academic progress and awareness of their educational status. The domain of regulation of situational interest investigated the students’ views on engaging in activities such as creating games, gamification, relating subjects to personal interests, and performing enjoyable tasks to increase their attraction, enjoyment, and interest in the learning environment. The self-consequating domain explored the students’ perspectives on making commitments to themselves to complete tasks or fulfill academic responsibilities and receive rewards or punishments. The domain of preventional situational awareness explored the students’ opinions on actions such as reverse modeling of negative role models or actively seeking information about their future educational prospects [19].

To gather data on the quality of clinical education, the Maastricht Clinical Teaching Questionnaire (MCTQ) developed by Stalmeijer et al. was utilized. This questionnaire, based on the cognitive apprenticeship model, was designed to assess the teaching skills of clinical instructors during medical clinical rotations [20, 21] translated MCTQ into Persian and evaluated it psychometrically [21]. The content validity ratio of the Persian version was confirmed to be 0.82, and the content validity index was 0.91. The reliability of the questionnaire was confirmed with a Cronbach’s alpha coefficient of 0.95. Confirmatory factor analysis results indicated a good fit for the 7-factor structure (21). The questionnaire consisted of 22 items categorized into 7 domains: 3 items in the modeling domain, 3 items in the coaching domain, 4 items in the scaffolding domain, 4 items in the articulation domain, 2 items in the reflection domain, 3 items in the exploration domain, and 3 items in learning environment domain. All items of the questionnaire were assessed using a 5-point Likert scale (ranging from strongly disagree to strongly agree).

### Data collection

This study was conducted in two stages including motivational self-regulation strategies in students before entering the clinical exposure phase and simultaneous measurement of students’ perception of clinical education and motivational self-regulation strategies at the end of the first semester of clinical exposure. In the first stage, at the beginning of the academic semester, the MSMQ was made available to students electronically. Two weeks later, a follow-up was conducted through social media to remind students who had not yet responded. In the second stage, the same group of students completed both the MSMQ and the MCTQ.

### Data analysis

After the completion of the questionnaires, the data was entered into the SPSS software. The Pearson correlation coefficient was used to determine the relationship between the dimensions of the cognitive apprenticeship model and motivational self-regulation strategies.

### Ethical consideration

This study was approved by the ethics committee of Kerman University of Medical Sciences (No: IR.KMU.REC.1400.703). Participants did not receive any incentives, and participation was voluntary. Informed consent for participation was obtained based on the proposal approved by the ethics committee. The participants were also assured of the confidentiality of their information, and it was explained that the results would only be used for research objectives.

## Results

A total of 55 medical students responded to the questionnaires (68.75%). 29 participants (52.7%) were female and 26 participants (47.3%) were male. The score for motivational self-regulation strategies of the students before entering the clinical exposure phase was M = 113.43, SD = 20.08 and it was M = 116.20, SD = 20.52 at the end of the first semester of clinical exposure. These findings show the students’ use of motivational self-regulation strategies has increased.

The mean and deviation of the MSMQ’s domains before entering clinical exposure were as follows regulation of the value (M = 24.17, SD = 4.07), Environmental structuring (M = 15.06, SD = 2.68), Regulation of relatedness (M = 13.59, SD = 3.33), Promotional situational awareness (M = 18.50, SD = 4.32), Regulation of situational interest (M = 12.44, SD = 3.40), Self-consequating (M = 11.98, SD = 3.80), Preventional situational awareness (M = 17.69, SD = 4.75).

The mean and deviation of the MSMQ’s domains at the end of the first semester of clinical exposure were as follows regulation of the value (M = 23.89, SD = 4.15), Environmental structuring (M = 14.89, SD = 2.64), Regulation of relatedness (M = 14.04, SD = 3.22), Promotional situational awareness (M = 19.37, SD = 4.49), Regulation of situational interest (M = 12.93, SD = 3.39), Self-consequating (M = 12.93, SD = 5.57), Preventional situational awareness (M = 18.11, SD = 4.97).

The findings showed that students utilized the regulation of the value strategy more frequently both before entering clinical exposure and at the end of the first semester of clinical exposure. Additionally, the self-consequating strategy had the lowest level of utilization before entering clinical exposure. While this strategy along with regulation of situational interest had the lowest level of utilization at the end of the first semester of clinical exposure. Table 1 provides descriptive information on the status of motivational self-regulation strategies of students before entering the clinical exposure phase and at the end of the first semester of clinical exposure.

**Table 1:** Status of motivational self-regulation strategies in medical students before entering clinical exposure and at the end of the first semester of clinical exposure

Motivational self-regulation strategies	Before entering clinical exposure		End of the first semester of clinical exposure	
	Mean	Std. Deviation	Mean	Std. Deviation
Regulation of the value	24.17	4.07	23.89	4.15
Environmental structuring	15.06	2.68	14.89	2.64
Regulation of relatedness	13.59	3.33	14.04	3.22
Promotional situational awareness	18.50	4.32	19.37	4.49
Regulation of situational interest	12.44	3.40	12.93	3.39
Self-consequating	11.98	3.80	12.93	5.57
Preventional situational awareness	17.69	4.75	18.11	4.97
Overall score	113.43	20.08	116.20	20.52

Results from the Pearson correlation test revealed significant correlations between modeling, coaching, scaffolding, reflection, exploration, learning environment, and motivational self-regulation strategies. In other words, as these dimensions improve, motivational self-regulation strategies among medical students increase. However, there was no significant correlation between the articulation dimension of the cognitive apprenticeship model and motivational self-regulation strategies (Table 2).

**Table 2:** Relationship between dimensions of the cognitive apprenticeship model and motivational self-regulation strategies

Variable	Pearson correlation	P-Value
Modeling	0.356	0.008
Coaching	0.342	0.011
Scaffolding	0.452	0.001
Articulation	0.148	0.282
Reflection	0.393	0.003
Exploration	0.271	0.046
Environmental learning	0.299	0.026

## Discussion

The present study aimed to investigate the role of clinical exposure on motivational self-regulation strategies of medical students based on the cognitive apprenticeship model. This model emphasizes the importance of learning experiences in real-world settings to promote the development of self-regulation skills. By examining the relationship between clinical exposure and motivational self-regulation skills based on the Cognitive Apprenticeship Model, this study contributes to our understanding of how educational experiences can shape the psychological attributes of medical students. The findings of this study revealed several important insights regarding the relationship between clinical exposure and motivational self-regulation skills.

Firstly, our results demonstrated a correlation between cognitive apprenticeship model dimensions including modeling, Coaching, Scaffolding, Reflection, and Exploration and motivational self-regulation skills in medical students, [22] stated that the self-regulation of motivation in students was improved by clinical faculty’ use of active teaching methods derived from learning theories, such as the cognitive apprenticeship model [22].

Furthermore, this study suggests that as medical students observing the behaviors of clinical faculty members during training and treatment, and engaging in everyday interactions contributed to the improvement of their ability to regulate their motivation and engagement in the learning process, [23] emphasized modeling as one of the methods for teaching motivational self-regulation strategies [23, 24] demonstrated a significant relationship between the presence of role models and higher levels of motivational self-regulation strategies [24]. Observations of medical students regarding behaviors, particularly the behaviors of their role models, have a greater impact on their learning compared to formal teaching [25]. Given the importance of this issue, numerous studies have made efforts to enhance the role of modeling through educational interventions, [26] demonstrated that role modeling has a significant influence on the professional behavior of medical students through a long-term faculty development program aimed at strengthening modeling among clinical instructors [26].

Our study results suggest that as students gain more exposure to clinical settings and real-world patient care, their ability to regulate their motivation and engagement in the learning process improves. This finding aligns with the principles of the Cognitive Apprenticeship Model, which posits that authentic experiences facilitate the development of self-regulatory skills. The results indicated that clinical exposure indirectly led to the enhancement of motivational self-regulation strategies for medical students. From the perspective of Bandura’s social cognitive theory, students’ ability to actively try to maintain or increase motivation in the face of motivational challenges in the form of motivational self-regulation is affected by various factors in the learning environment [14].

Our study indicated that specific aspects of the clinical and social contexts of patients may have differential effects on motivational self-regulation skills. For example, students who were provided with live visualization instead of just a theoretical approach and observe patient care take on increasing levels of responsibility and demonstrated higher levels of self-regulation. Both processes, by sharing theoretical foundations and practical interventions, can aid students in their goal-achievement, personal development, and overall progress. This integration can lead to a more comprehensive approach to supporting students in their growth and success [27]. [18] suggested that clinical faculty members establish a close and empathetic relationship with students to enhance motivation. This relationship leads to the improvement of the regulation of relatedness strategy, which aligns with our findings [18].

It is well-established that scaffolding has a positive impact on learners both cognitively and emotionally, influencing not only their skills and knowledge but also their motivation and self-confidence. It is reasonable to assume that most scaffolding designs intended for cognitive support also improve students’ motivation by increasing their expectations for success [27, 28] stated that both scaffolding and coaching are highly effective for learning in clinical settings [28]. This correlation aligns with the findings of the present study, suggesting the design of scaffolding interventions that support both motivation and learners’ cognition.

Articulation as part of the learning process can aid learners in motivational self-regulation by encouraging critical self-evaluation and maintaining motivation, [29] revealed that based on the principles of the cognitive apprenticeship model, articulation and encouraging students to reflect on their performance throughout the internship period are effective factors in clinical education. However, they found that certain environmental factors, such as time constraints, can limit the opportunity for articulation [29]. Therefore, considering that the present study revealed no significant relationship between articulation from the cognitive apprenticeship model and motivational self-regulation strategies, it is possible that time limitations from both the learner’s side (limited time in the clinical setting) and the clinical faculty member’s side (insufficient time for genuine observation and mentoring) could be the reasons behind the absence of a meaningful relationship.

Learners engage in reflection and make meaning of their knowledge by expressing their thoughts and arguments and increasing their metacognitive awareness. Reflection allows learners to analyze and revise their expressions, leading to better understanding and expertise. The significant relationship between reflection and motivational self-regulation strategies in this study is in line with [30] that suggested effective modeling, through creating a reflective space, generates a desire for learning in learners and, by fostering trust, enhances their interest and attention in learning and reflective practices [30].

Exploration is one of the basic requirements to facilitate self-motivation in personal development. The goal of exploration in the field of education is to encourage comprehensive independence in terms of problem-solving and formulating solutions [31]. In exploration, the learner is guided in the selection and evaluation of cognitive tasks, in identifying mistakes in the process of learning, and in choosing goals. Based on our results, there is a significant relationship between exploration and motivational self-regulation strategies, which can be effective in guiding learners in this type of learning process.

Some researchers consider motivational self-regulation to be inherently a social process, and they believe that self-regulation skills are developed and nurtured when learners participate in multiple social, cultural, and institutional environments [32]. According to this perspective and in support of the present findings, it seems necessary to pay more attention to conducting further studies and providing theoretical frameworks and specialized models in this field, considering the highly vital, complex, and diverse nature of medical sciences.

The significance of the impact of context on the process of meta-motivation has been highlighted by [13], specifically focusing on the contextual and social aspects of motivational self-regulation strategies. It therefore seems essential, considering the vital, complex, and diverse nature of medical sciences, to prioritize conducting further studies and providing theoretical frameworks and specialized models in this field.

Furthermore, our findings strongly challenge the notion that motivational self-regulation strategies don’t automatically develop in the clinical learning environment [33]. However, our results demonstrated that even without specific educational interventions and solely by placing students in a nurturing social environment, motivational self-regulation skills are enhanced in students.

This present research also has limitations. Firstly, it was conducted only in one medical science university, which may limit the generalizability of the findings. Replication studies in diverse medical education settings are warranted to validate and extend these results. This is especially important considering the findings of the current study, which indicate a significant relationship between the learning environment and the motivational self-regulation strategies of students. Secondly, as this study is an observational study aiming to examine the role of clinical exposure based on the cognitive apprenticeship model on motivational self-regulation strategies, it is suggested to conduct an interventional study in this regard. Also, observational and interventional studies are recommended to provide a more comprehensive assessment of motivational self-regulation skills.

## Conclusion

Clinical exposure plays a vital role in fostering medical students’ self-regulation abilities. Providing meaningful clinical exposure opportunities by medical educators and curriculum designers can facilitate the development of motivational self-regulation skills, ultimately enhancing students’ ability to navigate the complex challenges of medical education and practice.

## Data Availability

The datasets used and/or analyzed during the current study are available from the corresponding author upon reasonable request.
